# Biomarker study of symptomatic intracranial atherosclerotic stenosis in patients with acute ischemic stroke

**DOI:** 10.3389/fneur.2023.1291929

**Published:** 2023-12-06

**Authors:** Yingyue Ding, Jinjian Li, Huiyu Shan, Song Yang, Xiyuan Wang, Dexi Zhao

**Affiliations:** ^1^Department of Encephalopathy, The Affiliated Hospital of Changchun University of Traditional Chinese Medicine, Changchun, Jilin, China; ^2^School of Chinese Medicine, Changchun University of Chinese Medicine, Changchun, Jilin, China

**Keywords:** acute ischemic stroke, sICAS, recurrence, biomarkers, 4D label-free proteome quantification technology

## Abstract

**Objective:**

Acute ischemic stroke (AIS) is characterized by high rates of morbidity, disability, mortality, and recurrence, often leaving patients with varying degrees of sequelae. Symptomatic intracranial atherosclerotic stenosis (sICAS) is a significant contributor to AIS pathogenesis and recurrence. The formation and progression of sICAS are influenced by pathways such as lipid metabolism and inflammatory response. Given its high risk of clinical recurrence, timely assessment of intracranial vascular stenosis in AIS is crucial for diagnosing sICAS, treating stroke, and preventing stroke recurrence.

**Methods:**

Fourteen AIS patients were divided into stenosis and control groups based on the presence or absence of intracranial vessel stenosis. Initially, 4D Label-free proteome quantification technology was employed for mass spectrometry analysis to identify differential proteins between the groups. Subsequently, functional enrichment analysis, including GO classification, KEGG pathway, and Domain, revealed trends related to differential proteins. The STRING (v.11.5) protein interaction network database was used to identify differential protein interactions and target proteins. Finally, parallel reaction monitoring (PRM) validated the selected target proteins.

**Results:**

Mass spectrometry identified 1,096 proteins, with 991 being quantitatively comparable. Using a *p*-value <0.05 and differential expression change thresholds of >1.3 for significant up-regulation and < 1/1.3 for significant down-regulation, 46 differential proteins were identified: 24 significantly up-regulated and 22 significantly down-regulated. PRM experiments validated five proteins related to lipid metabolism and inflammatory response: namely alpha-2-macroglobulin (A2M), lipopolysaccharide-binding protein (LBP), cathepsin G (CTSG), cystatin (CST)3, and fatty acid-binding protein (FABP)1.

**Conclusion:**

The detection of changes in these five proteins in AIS patients can aid in the diagnosis of sICAS, inform stroke treatment, and assist in preventing stroke recurrence. Moreover, it can contribute to the development of drugs for preventing AIS recurrence by integrating traditional Chinese and Western medicine.

## Introduction

AIS is the predominant form of stroke, constituting 60–70% of all stroke cases (1, 2). With an annual incidence reaching 12 million globally, China experiences the highest incidence at 3.83 million cases annually and an annual recurrence rate of 14.7% (3). sICAS is a significant contributor to the onset and recurrence of ischemic stroke (4–7). sICAS is characterized by the narrowing of intracranial arteries due to atherosclerosis, accompanied by a history of ischemic stroke or transient ischemic attack (TIA) in the region supplied by the constricted artery. In China, sICAS is responsible for 46.6% of AIS cases (8). Atherosclerosis is recognized as a chronic systemic inflammatory disease, with intracranial arteries being the principal site of manifestation. The pathological progression ranges from endothelial damage to the formation of lipid streaks in the arterial wall, which are visible to the naked eye. Under the influence of *in vivo* inflammatory factors, these lipid streaks progressively evolve into atheromatous plaques. As the condition advances, vessel wall remodeling occurs, and the plaque encroaches upon the vessel lumen. This can eventually lead to vessel narrowing or even plaque rupture or detachment, resulting in complete occlusion of the vessel lumen and triggering acute cardiovascular and cerebrovascular events. The pathogenesis of atherosclerosis encompasses various theories, including lipid infiltration, injury response, inflammation, and a combination of genetic and environmental factors.

Currently, the optimal treatment approach for sICAS remains uncertain. Two significant randomized controlled trials, namely the SAMMPRIS (Stenting and Aggressive Medical Management for Preventing Recurrent Stroke in Intracranial Stenosis) trial (9) and the VISSIT (vitesse intracranial stent study for ischemic stroke therapy) trial (10), did not establish the superiority of endovascular stenting over intensive drug therapy. Despite intensive drug therapy, 4–20% of patients with sICAS experience recurrent stroke annually (11–13). sICAS is associated with a high risk of clinical recurrence. For instance, the annual recurrence rate of ischemic stroke in the region of the stenotic vessel is approximately 8% in patients with intracranial segmental stenosis of the internal carotid artery (ICA) (14). The WASID study reported high clinical recurrence rates in patients with sICAS in the basilar artery (BA) region, with symptomatic BA, intracranial vertebral artery (VA) and posterior cerebral artery (PCA) symptomatic stenoses showing stroke recurrence rates of 10.7, 7.8, and 6%, respectively (15). The annual incidence of ischemic stroke in any vascular region was 15, 13.7, and 6%, respectively (15). Thus, a timely and effective assessment of intracranial vascular stenosis is crucial for preventing recurrent ischemic stroke. In recent years, biomarkers have emerged as a focal point of research due to their rapid, effective, and non-invasive nature. Given this context, this study is the first to explore changes in plasma proteins in sICAS patients with acute ischemic stroke.

Utilizing 4D Label-free proteome quantification technology, this study identified unique differential proteins closely related to sICAS in acute ischemic stroke patients. Our findings indicate that several proteins contribute to the formation and development of sICAS in these patients. Five proteins—A2M, LBP, CTSG, CST3, and FABP1—were further validated using PRM. These biomarkers, implicated in lipid metabolic pathways and inflammatory response processes, can aid in the diagnosis of sICAS, treatment of stroke, and prevention of stroke recurrence. Additionally, they may facilitate the development of drugs that prevent the recurrence of acute ischemic stroke by integrating both traditional Chinese and Western medicine approaches.

## Materials and methods

### Patient inclusion and exclusion criteria and serum collection

#### Patient inclusion and exclusion criteria

This research is a retrospective study. The inclusion criteria for patients were as follows: 1. Diagnosis of acute cerebral infarction as per the Chinese Guidelines for the Diagnosis and Treatment of Acute Ischemic Stroke 2018, confirmed through patient history and Computed Tomography (CT)/Magnetic Resonance Imaging (MRI) examination, and their vascular condition was assessed by head Magnetic Resonance Angiography (MRA). 2. Classification as either large artery atherosclerotic or small artery occlusive cerebral infarction according to the trial of Org10172 in acute stroke treatment (TOAST). 3. Age between 50 and 70 years. 4. Ability to cooperate with baseline data collection. 5. Informed consent form signed by the patient or legal guardian. A total of 14 patients meeting these criteria were included and divided into two groups: 7 cases in the stenosis group (XXZ) and 7 cases in the control group (DZZ), based on the presence or absence of intracranial vessel stenosis. The stenosis group included cases with MRA evidence of intracranial vascular stenosis supplying the associated infarct area, while the control group included cases with MRA evidence of intracranial atherosclerotic changes or no significant abnormalities.

The exclusion criteria were as follows: 1. Diagnosis of transient ischemic attack (TIA), hemorrhagic stroke, or mixed stroke; 2. TOAST classification of cardiogenic embolic, other definite etiology, or ischemic stroke of unknown origin; 3. Diagnosis of cerebral infarction with unconsciousness; 4. Inability to cooperate in clinical data collection. Given that intracranial atherosclerosis has numerous secondary causes and necessitates control over multiple variables, this study does not proceed with these investigations at this time.

#### Serum sample collection

The procedure for serum collection is as follows: 1. Fasting peripheral blood (approximately 5 mL) is collected from all patients into ethylene diamine tetraacetic acid (EDTA) anti-coagulated tubes and immediately mixed by gently inverting 5–6 times; 2. The samples are left to stand for 30–45 min at 4°C; 3. The samples are then centrifuged at 1,300 g for 10 min at 4°C; 4. The upper layer of serum (typically a clear yellow liquid) is transferred to a centrifuge tube using a pipette; 5. The sample is immediately frozen at −80°C. Caution: 1. Samples displaying hemolysis should be rejected; 2. Serum separation should be carried out promptly, and repeated freezing and thawing after separation should be avoided.

### Research content

Fourteen patients with acute ischemic stroke who met the inclusion criteria were classified into XZZ and DZZ groups based on the presence or absence of intracranial vessel stenosis. Initially, 4D Label-free proteome quantification technology was utilized to perform mass spectrometry analysis on all patients, identifying differential proteins between the two groups. Subsequently, functional enrichment analysis using GO classification, KEGG pathway, domain, and other bioinformatics libraries was conducted to ascertain trends related to differential proteins. Additionally, the STRING (V.11.5) protein interaction network database was employed to compare and identify differential protein interactions and corresponding target proteins. Finally, PRM was used to validate the selected target proteins.

### Statistical analysis

SPSS 25 software was employed for the statistical analysis of baseline data. Descriptive statistics for demographic information included calculations for continuous variables’ number of cases, means, standard deviations, minimum, and maximum values; frequency and composition ratios were calculated for count and rank information. Depending on data normality, either the t-test or rank sum test was used to compare differences between groups for measurement data, while the chi-square test or Fisher’s exact probability method was used for count data.

For differential protein screening, a threshold of change >1.3 and a *p* value <0.05 were set. Firstly, samples for comparison were selected, and the ratio of the mean relative quantitative values of each protein in multiple replicate samples was used as the Fold Change. Secondly, to determine the significance of differences, the relative quantitative values of each protein in the comparison group samples were subjected to a t-test, and the corresponding *p* value was calculated. The default p value was set at <0.05. Finally, to ensure that the test data conformed to the normal distribution required by the t-test, the relative quantitative protein values were Log2 transformed before testing. An analysis of variance was used to identify significant changes when the *p* value <0.05, with a change in differential expression of more than 1.3 set as the threshold for significant up-regulation and less than 1/1.3 for significant down-regulation.

### Patient baseline conditions

Baseline data from both groups, including age, National Institute of Health Stroke Scale (NIHSS), Barthel Index (BI), modified Rankin Scale (mRs), gender, and smoking history, were statistically analyzed using SPSS25 software to assess the comparability of the two groups.

### Proteomics analysis

#### Protein extraction

Samples were retrieved from −80°C storage and centrifuged at 12,000 g at 4°C for 10 min. Subsequently, cell debris was discarded, and the supernatant was transferred to a fresh centrifuge tube. High-abundance proteins were removed using the Pierce™ Top 14 Abundant Protein Depletion Spin Columns Kit (Thermo Scientific). Protein concentration was ascertained using the BCA kit.

#### Trypsin digestion

Equal amounts of protein from each sample were digested. The volume was standardized with lysis solution, followed by the addition of dithiothreitol (DTT) to a final concentration of 5 mM for 30 min reduction at 56°C. Iodoacetamide (IAM) was then added to a final concentration of 11 mM, and samples were incubated for 15 min at room temperature in the dark. Urea was replaced thrice with 8 M urea and subsequently thrice with replacement buffer. Trypsin was added at a 1:50 ratio (protease:protein, m/m) for overnight digestion. Peptides were recovered by centrifuging at 12,000 g for 10 min at room temperature. Peptides were also recovered once with ultrapure water, and both peptide solutions were combined.

#### Liquid chromatography-mass spectrometry analysis

An Orbitrap Exploris™ 480 (ThermoFisher Scientific) mass spectrometer was employed for analysis. The ion source voltage was set to 2.3 kV, and the FAIMS compensation voltages (CV) were set to −70 and −45 V. Both the peptide parent ion and its secondary fragments were detected and analyzed using a high-resolution Orbitrap. The primary mass spectrometry (MS) scanning range was set to 400–1,200 m/z, and the scanning resolution was set to 60,000; the secondary MS scanning range was fixed starting at 110 m/z, and the resolution of the secondary scan was set to 30,000, with the TurboTMT setting set to Off. The data acquisition mode used a data-dependent scanning (DDA) procedure, that is, after a primary scan, the top 15 peptide parent ions with the highest signal intensity were selected to enter the HCD collision cell sequentially, fragmented using 27% fragmentation energy, and similarly sequentially analyzed by secondary mass spectrometry. To optimize mass spectrometry utilization, the automatic gain control (AGC) was set to 7.5E4, the signal threshold was set to 1E4 ions/s, the maximum injection time was set to 100 ms, and the dynamic exclusion time for tandem mass spectrometry scans was set to 30 s to avoid repeated scans of the parent ions.

### Sample repeatability experiment

Pearson’s Correlation Coefficient (PCC) statistical analysis was utilized to assess the reproducibility of the project.

### Differential protein screening

Volcano plots were employed to display the results of differential protein screening. The horizontal axis represents the value of the ratio of differential expression change between XZZ and DZZ after Log2 transformation, while the vertical axis represents the value of the statistical test T-test value of p after −Log10 transformation. In the plot, red dots indicate proteins that are significantly up-regulated in XZZ relative to DZZ, blue dots indicate significant down-regulation, and gray dots indicate no significant differential regulation.

### Functional enrichment analysis of differential proteins

Enrichment analysis of differentially expressed proteins in XZZ and DZZ was performed at the levels of GO classification, KEGG pathway, and Domain. Fisher’s exact test was used to calculate the significance *p* value, aiming to determine whether the differentially expressed proteins exhibit a significant enrichment trend in certain functional types.

### Protein interaction network analysis

Differential protein database numbers or protein sequences obtained from the comparison group, based on the multiplicity of differences 1.3 screening, were extracted to identify differential protein interactions. These were compared with the STRING (v.11.5) protein interactions network database, using a confidence score > 0.7 (high confidence). The differential protein interactions were visualized using the R package “networkD3” tool.

### PRM validation for proteomics

PRM validation for proteomics included protein extraction, trypsin digestion, liquid chromatography–tandem mass spectrometry (LC–MS/MS) concatenated analyses, MaxQuant (V1.6.15.0) database searches, and data processing by Skyline 21.2 software. LC–MS/MS is a tandem mass spectrometry method that allows for simultaneous acquisition of both the molecular ion peak and the fragmentation ion peaks, facilitating quantitative and qualitative analysis.

## Results

### Baseline data situation

As depicted in Table 1 (data of measurement) and Table 2 (data of counting) statistics, no statistically significant differences were observed in age, NIHSS, BI, mRS, gender, or smoking history between the two groups, XZZ and DZZ (*p* > 0.05), indicating comparability between the groups.

**Table 1:** Baseline data status.

Indicators	Group	Case	Mean	Standard deviation	Minimum value	Maximum value	*Z*	*P-*value
Age	XZZ	7	57.14	7.11	51.00	69.00	0.026	0.874>0.05
	DZZ	7	57.57	7.93	50.00	69.00		
NIHSS	XZZ	7	2.43	2.15	1.00	7.00	−1.575	0.128>0.05
	DZZ	7	2.00	4.04	0.00	11.00		
BI	XZZ	7	81.43	31.72	20.00	100.00	−0.597	0.620>0.05
	DZZ	7	89.29	26.21	30.00	100.00		
mRs	XZZ	7	1.43	1.27	0.00	4.00	0.373	0.805>0.05
	DZZ	7	1.29	1.25	0.00	4.00		

The SPSS25 software was used for statistical analysis of baseline data (data of measurement).

**Table 2:** Baseline data status.

Indicators	Classification	XZZ		DZZ		*P*-value
		Case	Ratio of composition (%)	Case	Ratio of composition (%)	
Gender	Female	3	42.86	2	28.57	1.000>0.05
	Male	4	57.14	5	71.43	
Smoke	Yes	3	42.86	4	57.14	1.000>0.05
	No	4	57.14	3	42.86	

The SPSS25 software was used for statistical analysis of baseline data (data of counting).

### Protein identification results

**Figure 1:**
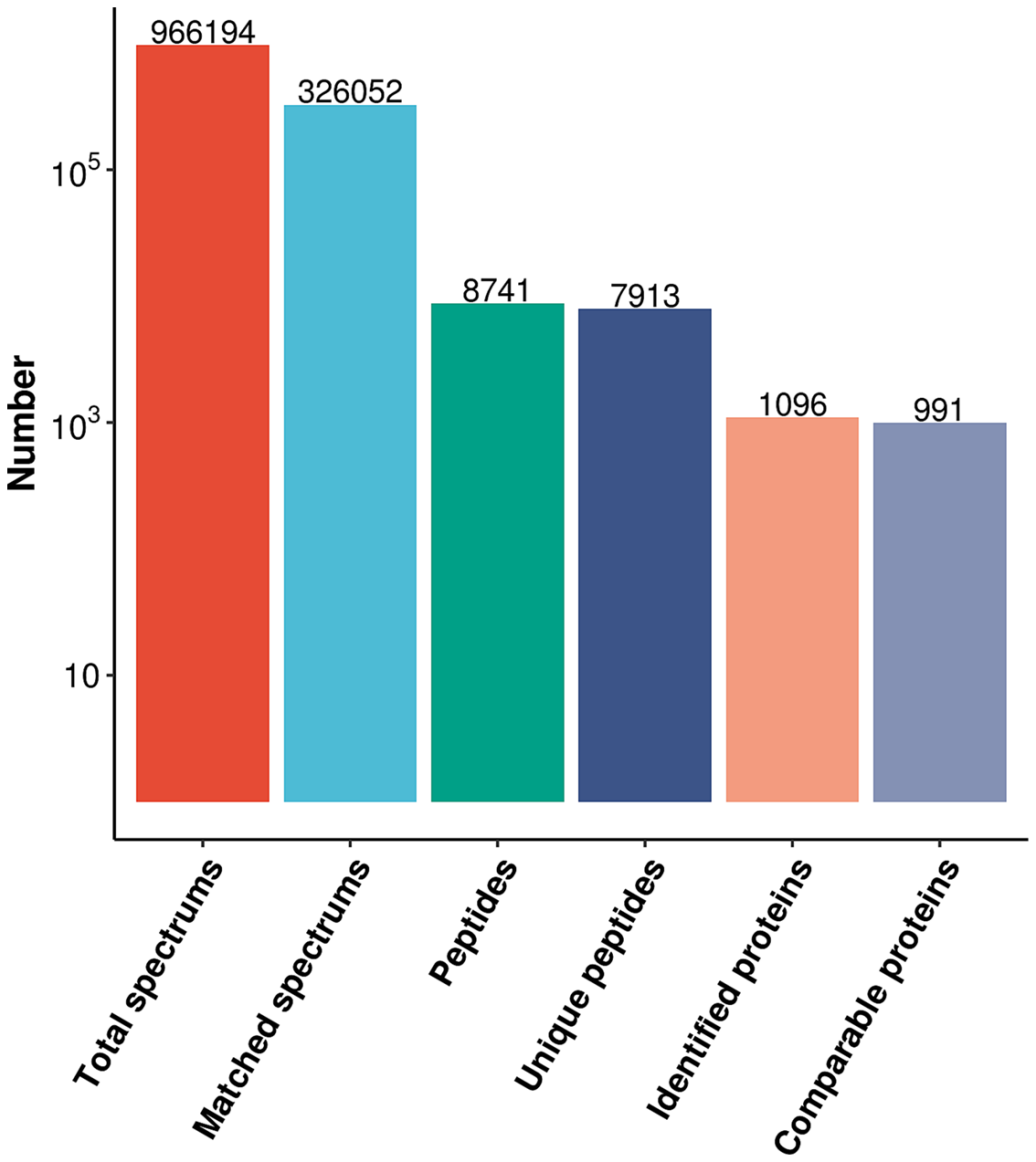
Protein identification results.

Mass spectrometry analysis was conducted to detect the protein profiles of XZZ and DZZ. The analysis yielded 966,194 secondary spectra numbers, of which 326,052 were valid spectra; 8,741 peptides, of which 7,913 were specific peptides; and 1,096 proteins, of which 991 could be quantitatively compared (Figure 1).

### Distribution and variability of protein intensity values

**Figure 2:**
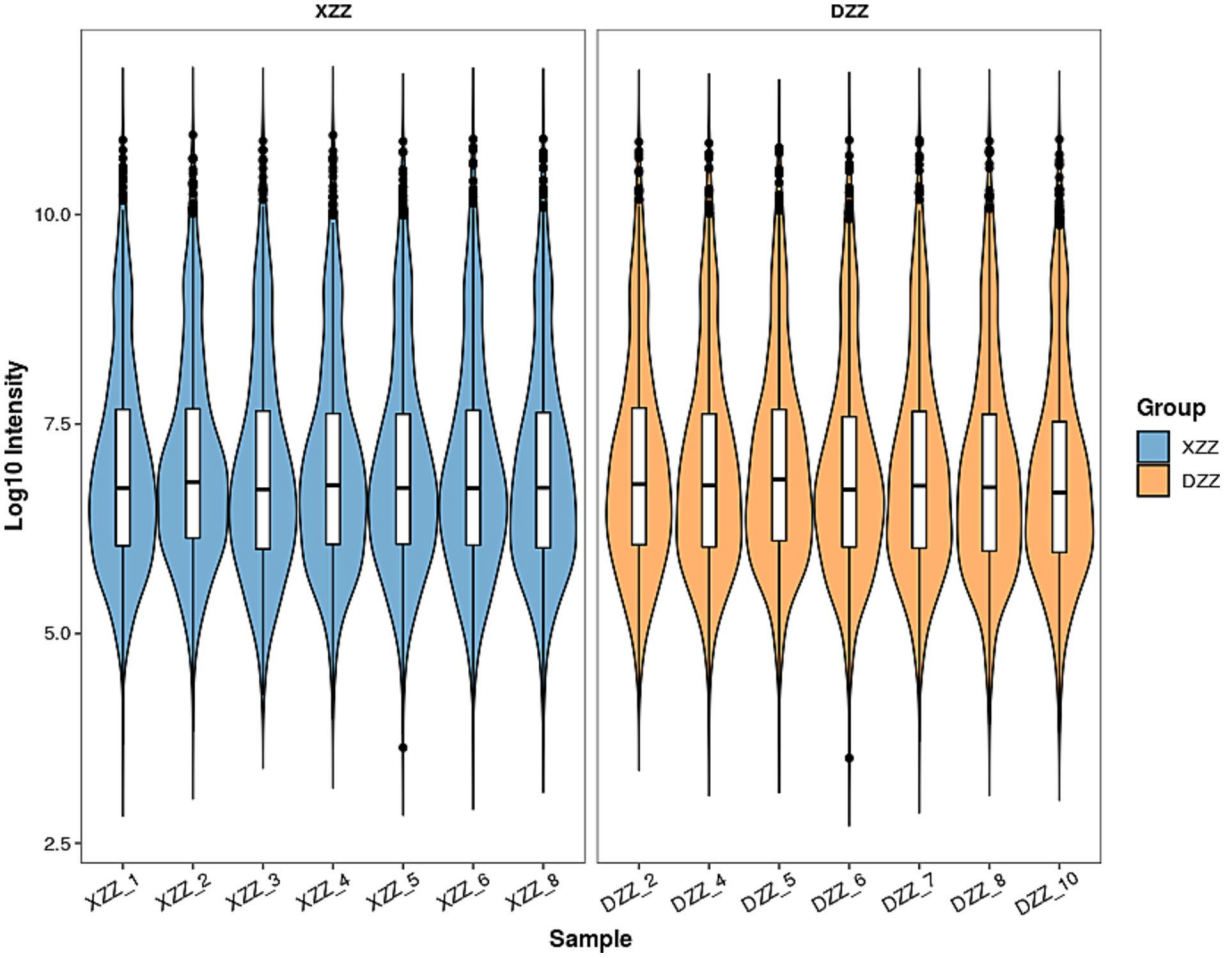
Distribution and difference of protein intensity values.

To explore the distribution and variability of protein intensity values among different samples, the protein intensity value of each sample was extracted and depicted using violin plots. The horizontal axis represents the sample name, while the vertical axis represents the intensity value after Log10 conversion. The color of the violin denotes different groupings. The inner part of the violin plot displays the box-and-line plot, with the box representing the middle 50% of the distribution interval of the group’s data. The outer part is the kernel density plot, where a larger area indicates a higher probability of the corresponding value’s distribution. Cross-sectional comparisons provide an indication of the data distribution’s dispersion within and between groups. If the sample means are at the same level, the sample quality is deemed good. The results indicate that the sample quality of this study is satisfactory (Figure 2).

### Sample repeatability experimental results

**Figure 3:**
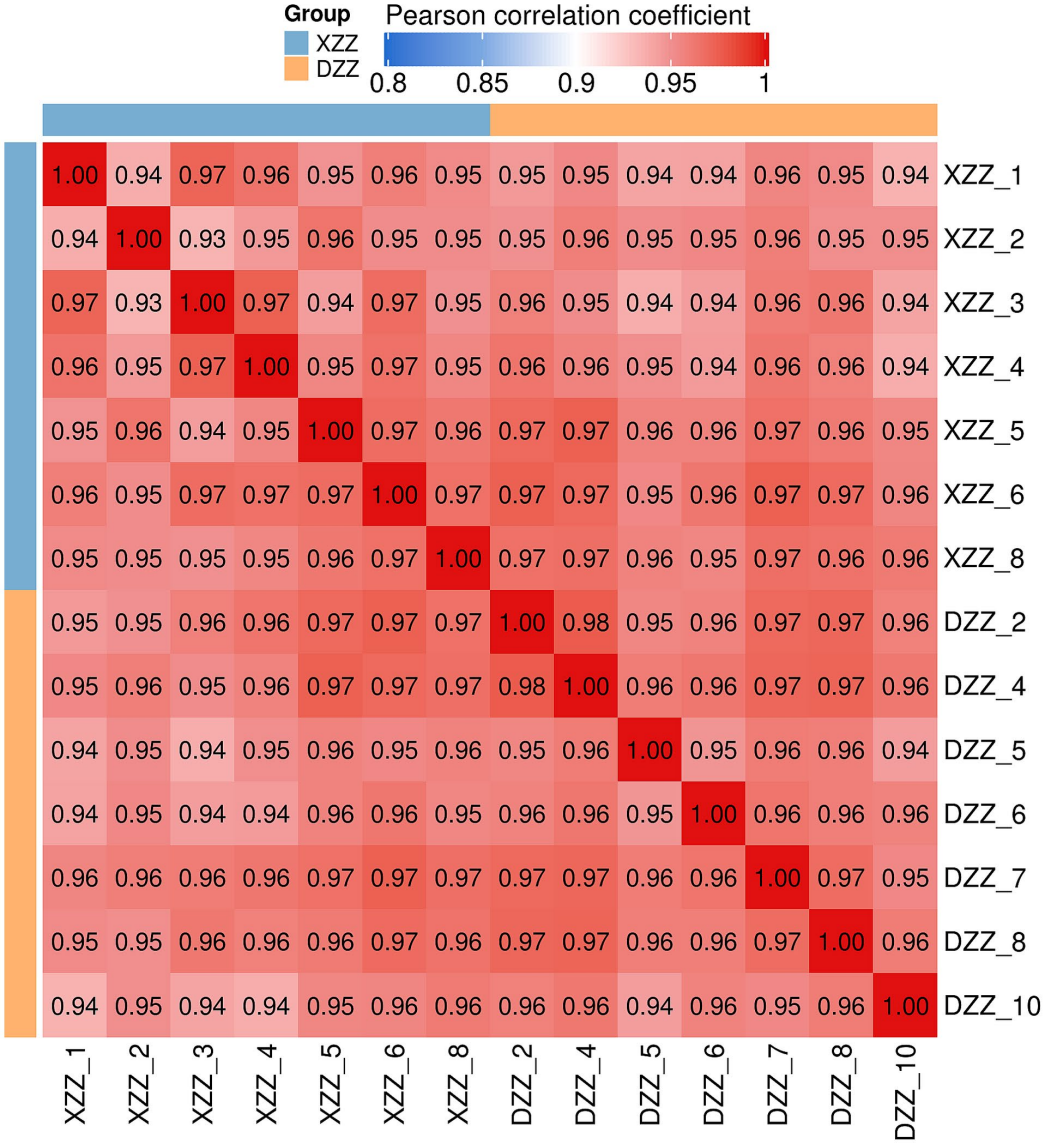
Sample repeatability test results.

A heatmap was plotted using Pearson’s correlation coefficient for pairwise comparisons of all samples of XZZ and DZZ. This coefficient measures the degree of linear correlation between two sets of data: a coefficient close to −1 indicates a negative correlation, close to 1 indicates a positive correlation, and close to 0 indicates no correlation. The analysis results show that the intra-group correlation for all samples is above 0.93 (Figure 3).

### Results of differential protein screening

**Figure 4:**
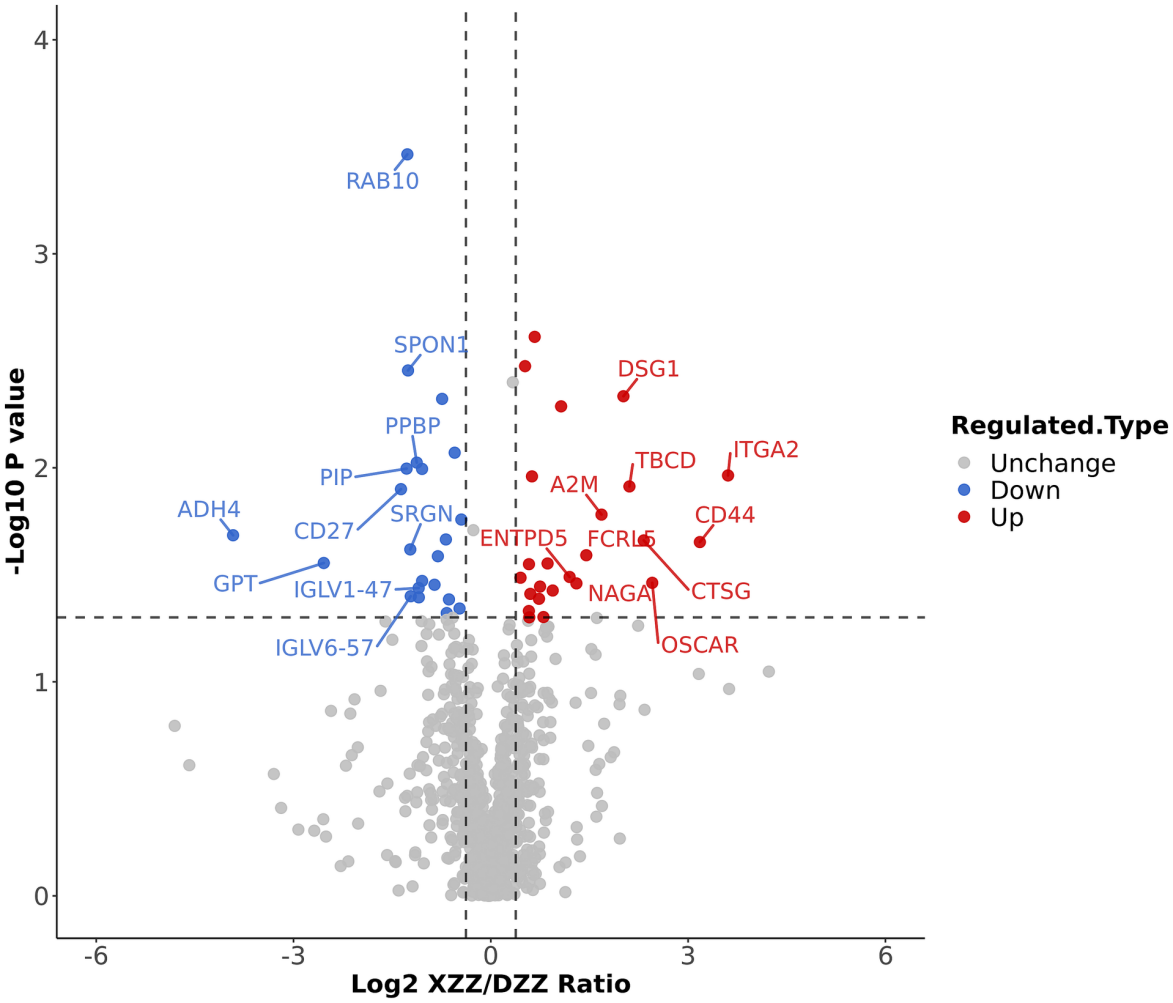
Differential protein screening results: The information of the Top10 differential proteins was marked down in the figure.

When the *p* value <0.05, a change threshold of differential expression greater than 1.3 was considered as significant up-regulation, and less than 1/1.3 was considered as significant down-regulation. Consequently, 46 differential proteins were identified, with 24 proteins significantly up-regulated and 22 proteins significantly down-regulated. The information about the top 10 up-regulated and down-regulated differential proteins (excluding points outside the threshold) is labeled in Figure 4. Ten proteins, immunoglobulin lambda variable (IGLV)6–57, IGLV1-47, glutamate pyruvic transaminase (GPT), alcohol dehydrogenase (ADH)4, cluster of differentiation (CD)27, prolactin-inducible protein (PIP), Serglycin (SRGN), pre-platelet basic protein (PPBP), Spondin-1 (SPON1), ras-associated binding (RAB10) were significantly down-regulated; 10 proteins such as A2M, Fc receptor-like protein 5(FCRL5), CD44, osteoclast-associated immunoglobulin-like receptor (OSCAR), tubulin-specific chaperone D (TBCD), ectonucleoside triphosphate diphosphohydrolase 5(ENTPD5), alpha-N-acetylgalactosaminidase (NAGA), desmoglein-1(DSG1), CTSG, integrin alpha (ITGA2) were significantly up-regulated.

### Results of functional enrichment analysis of differential proteins

**Figure 5:**
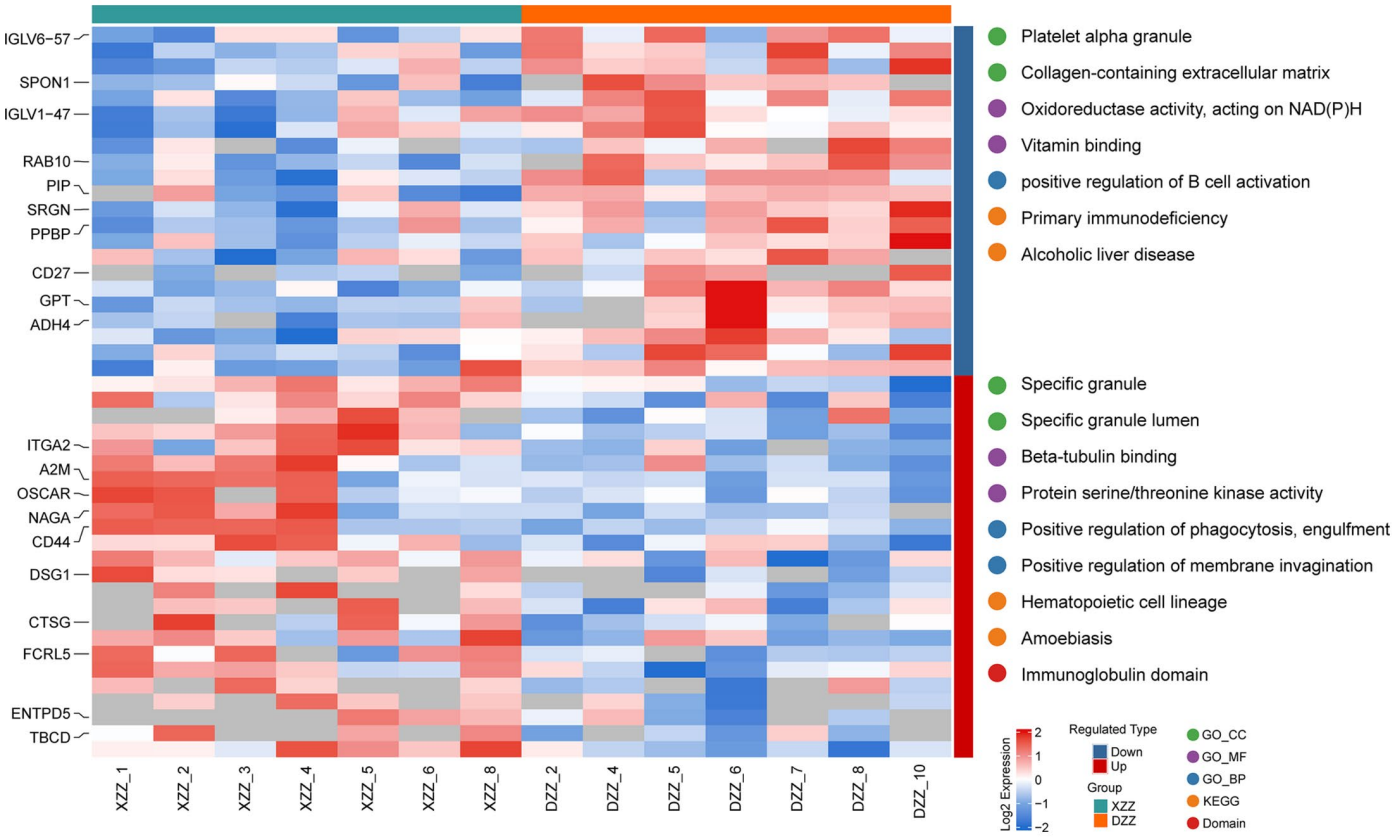
Results of functional enrichment analysis of differential proteins.

Functional enrichment analysis was conducted on all differential proteins using GO classification, the KEGG pathway, and the Domain Bioinformatics library. The results are depicted in Figure 5 heatmap. The horizontal coordinate represents the patient number, the vertical coordinate on the left side represents the top 10 up-regulated and down-regulated differential protein names, and the right side represents the top 3 pathways enriched by each bioinformatics library.

### Results of protein interaction network analysis

**Figure 6:**
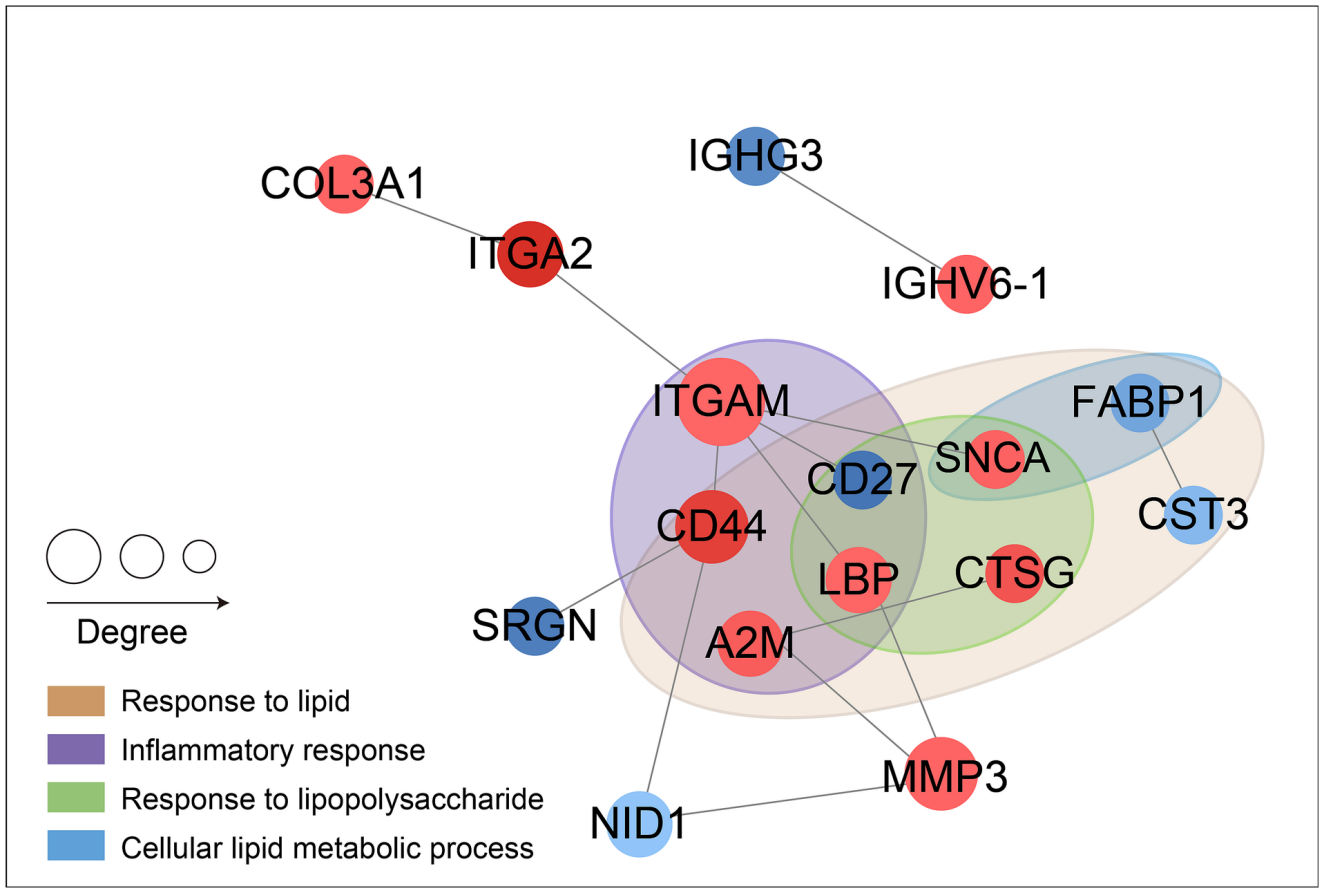
Results of protein interaction network analysis.

The lipid metabolic pathway and the inflammatory response pathway were given extra attention during the differential protein interaction network analysis. This was done as part of the functional enrichment analysis of differential proteins. The analysis primarily identified enrichment in four pathways: “inflammatory response,” “response to lipopolysaccharide,” “cellular lipid metabolic process,” and “response to lipid.” The results are depicted in Figure 6, where circles represent the differential proteins. Different colors indicate the differential expression of the proteins (blue for down-regulated proteins, red for up-regulated proteins), and the size of the dots signifies the degree of relationship with interacting proteins. Proteins integrin alpha-M (ITGAM), CD44, CD27, A2M, LBP are associated with “inflammatory response”; proteins alpha-synuclein (SNCA), CD27, LBP, CTSG are associated with “response to lipopolysaccharide”; proteins SNCA, FABP1 are related to the “cellular lipid metabolic process”; proteins FABP1, CST3, SNCA, CTSG, LBP, CD27, CD44, A2M are related to the “response to lipid.”

### PRM validation

**Figure 7:**
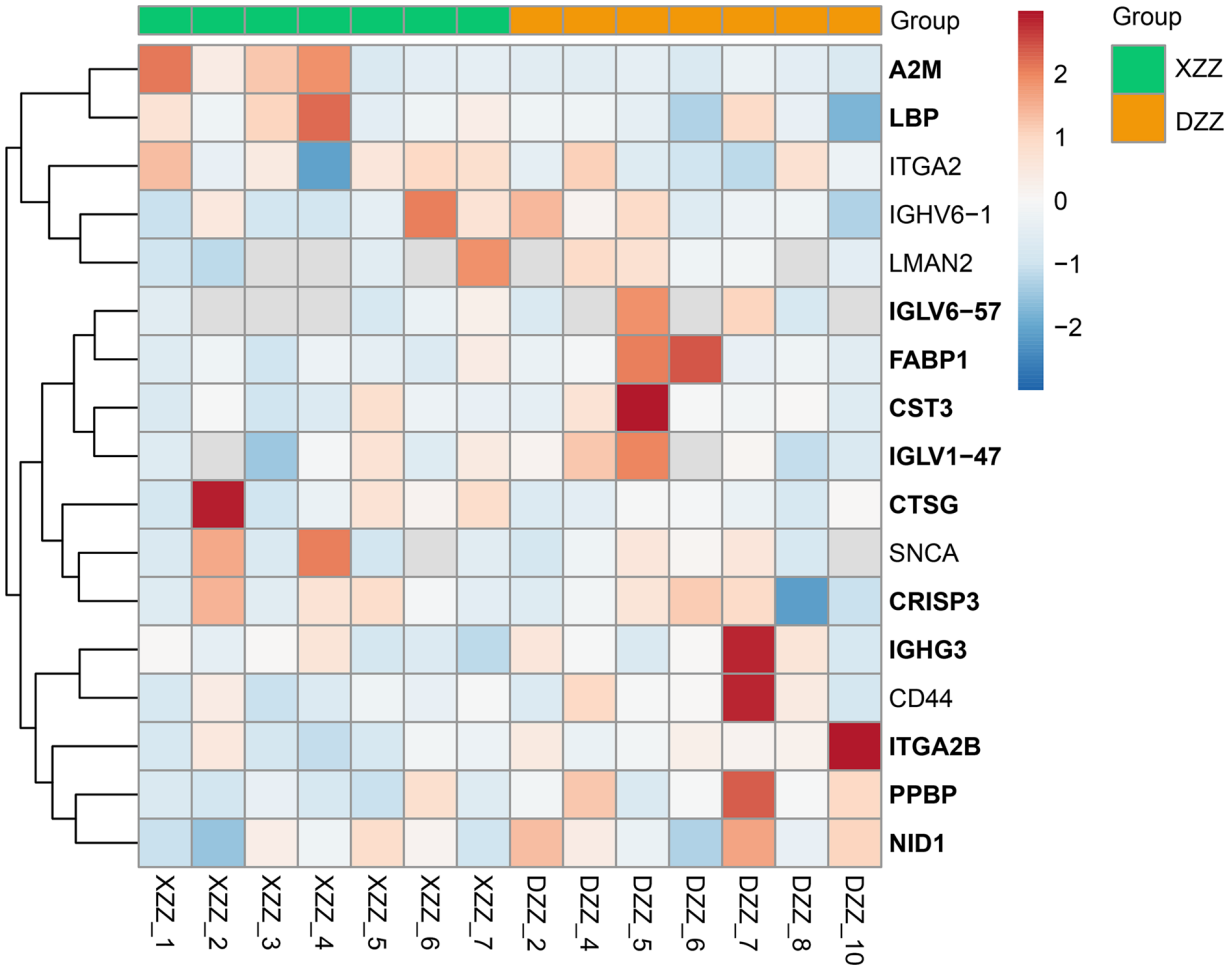
PRM heat map results. The x-coordinate indicates the sample numbers of XZZ and DZZ. The darker the color of the red module, the higher the expression, and the darker the color of the blue module, the lower the expression. The ordinate indicates the name of the protein, and the bold protein is consistent with the results of proteomics.

**Figure 8:**
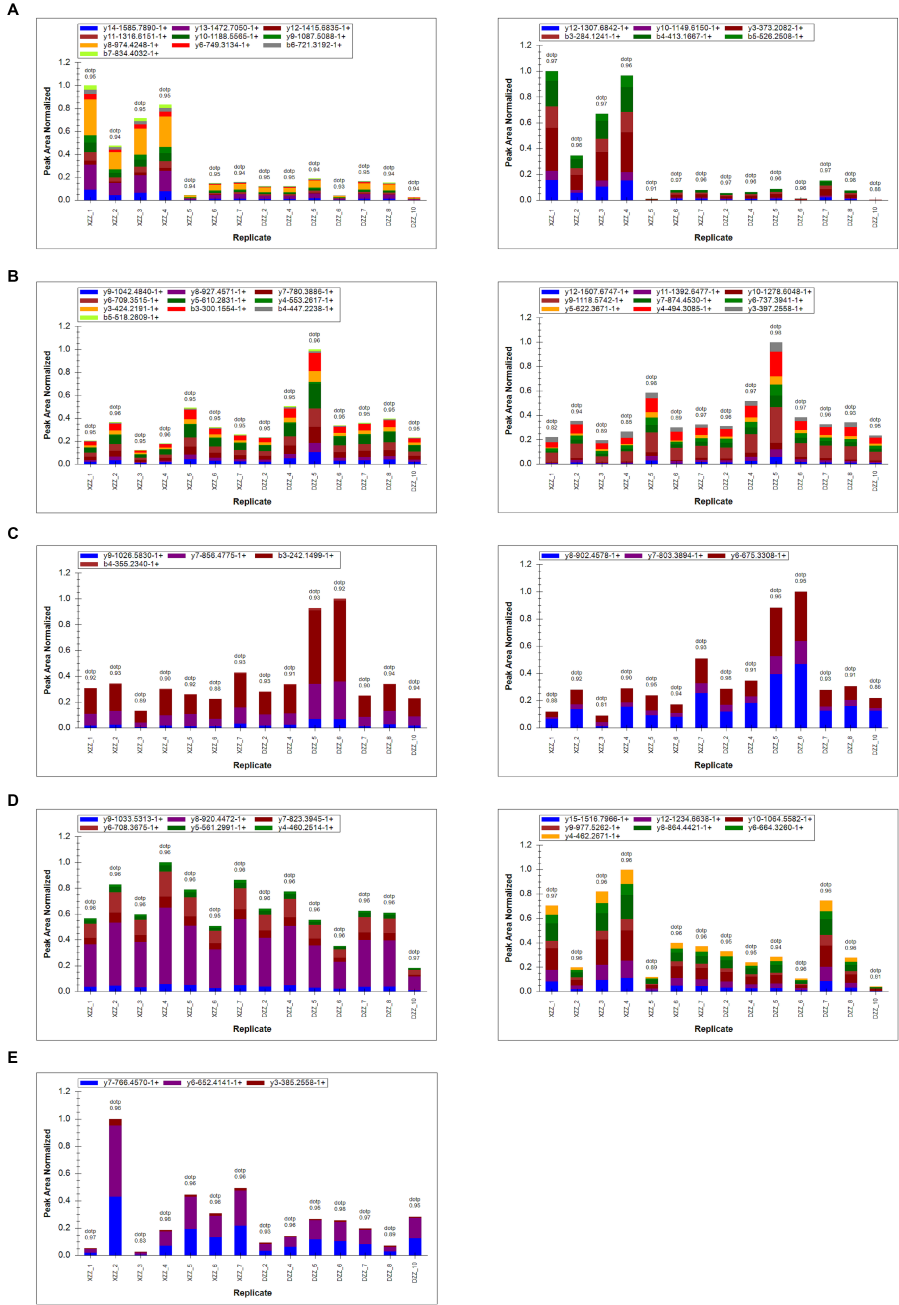
**(A–E)** Represent the fragment ion peak area distribution of protein A2M (P01023), CST3 (P01034), FABP1 (P07148), LBP (P18428), and CTSG (P08311), respectively.

PRM quantification is conducted using peak areas. In this experimental design, each protein was quantified using two unique peptides, though some proteins were identified with only one peptide due to sensitivity and other factors. This study selected 20 proteins related to the research for PRM validation. The heatmap results indicated that a total of 17 proteins were identified, 12 of which aligned with the proteomics results (Figure 7). Among these, five proteins are most pertinent to the lipid metabolism pathway and the inflammatory response pathway. Consequently, it was determined that, compared with DZZ, the proteins A2M, LBP, and CTSG were significantly up-regulated in XZZ, while the proteins CST3 and FABP1 were significantly down-regulated. The distribution of the peak area of fragment ions in these five proteins is presented in Figure 8.

## Discussion

**Figure 9:**
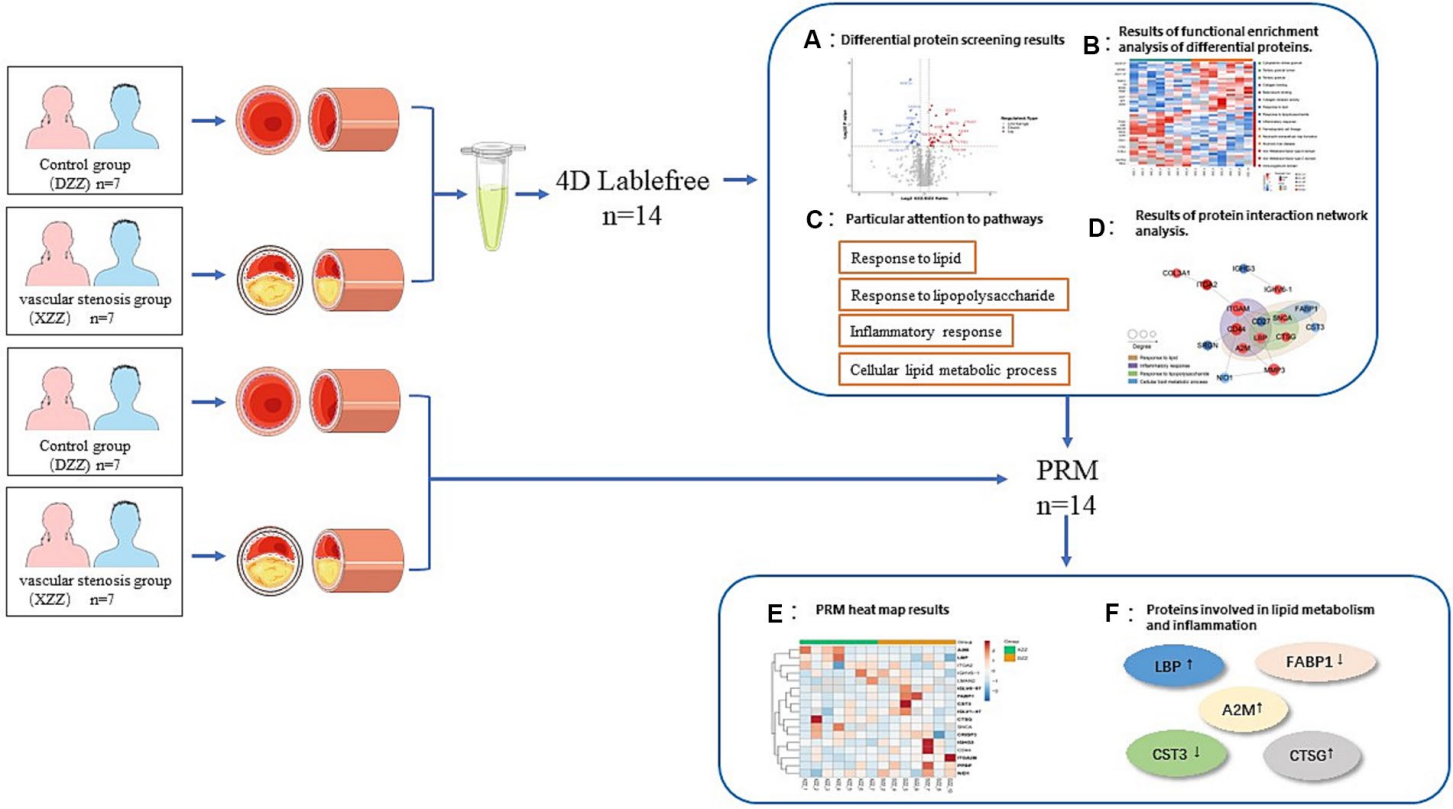
Technical roadmap. See section “Materials and methods” and “Results” for details of the experimental procedures.

In recent years, the release of guidelines for the prevention and treatment of acute ischemic stroke has led to substantial advancements in understanding its pathophysiological mechanisms, diagnosis, and treatment. However, limitations persist. sICAS is a significant contributor to the onset and recurrence of ischemic stroke. Although head MRA/head Computed Tomography Angiography (CTA)/head Digital Subtraction Angiography (DSA) can accurately assess intracranial stenosis, these methods are time-consuming and costly. Currently, no specific biomarkers for sICAS have been identified. In light of this, the present study included 14 patients aged 50–70 years, excluding those with cardiogenic embolicity, as atherosclerosis is closely related to cardiovascular disease development (16), and interfering factors should be excluded as much as possible. We limited the age range of participants to 50–70 years because this demographic is most commonly affected by acute ischemic stroke. Individuals under 50 are more prone to cardiovascular diseases (17). By doing so, we aimed to eliminate confounding factors from comorbid diseases and age-related variables, thus enabling a more precise prediction of protein expression differences. These predictions are crucial for identifying key factors and biological targets associated with the development of sICAS. The workflow of our study is depicted in Figure 9.

The objective of this study was to identify unique differential proteins closely related to sICAS in patients with acute ischemic stroke using a 4D proteomics approach. A total of 1,096 differential proteins were identified through proteomic analysis of plasma from these patients, with 991 proteins being quantitatively comparable. Peripheral blood, being easily accessible, detectable, and preservable, has been extensively used for assessing common clinical conditions (18–21). The analysis primarily enriched four pathways: Response to lipid, Response to lipopolysaccharide, Inflammatory response, and Cellular lipid metabolic process, all of which are associated with lipid metabolism and inflammatory responses. Protein interaction network analyses led to several conclusions: proteins ITGAM, CD44, CD27, A2M, and LBP are associated with “inflammatory response”; SNCA, CD27, LBP, and CTSG are linked to “response to lipopolysaccharide”; SNCA and FABP1 relate to “cellular lipid metabolic process”; and FABP1, CST3, SNCA, CTSG, LBP, CD27, CD44, and A2M are associated with “response to lipid.” Post-PRM validation, 12 proteins of interest aligned with the proteomics results, with 5 being highly relevant to lipid metabolism pathways and inflammatory response pathways (results shown in Figure 7). This study suggests that the differential expression of these five proteins—A2M, LBP, CTSG, CST3, and FABP1—can serve as markers for the diagnosis and assessment of sICAS. Compared to the DZZ group, proteins A2M, LBP, and CTSG were significantly up-regulated in the XZZ group, while CST3 and FABP1 were significantly down-regulated. Notably, this study is the first to reveal the association between these five proteins and symptomatic intracranial atherosclerotic stenosis in patients with acute ischemic stroke, also indicating potential relevance to other neurological diseases.

A2M is a crucial protease inhibitory molecule synthesized by astrocytes and neurons in the brain and serves as an acute-phase protein in the brain’s inflammatory response (22). Notably, A2M is markedly elevated in neuronal protrusions in senile plaques, neurofibrillary tangles, and atrophic neurons in Alzheimer’s disease patients’ brains (23). A2M is strongly associated with neurological disorders associated with dopaminergic neuronal dysfunction and cerebrospinal fluid (CSF) biomarkers within the coding region of Alzheimer’s disease (AD) susceptibility genes (22, 24). This study indicates that A2M, in the XXZ group of patients with acute ischemic stroke, is primarily associated with “inflammatory response” and “response to lipid.” Decreasing A2M expression may influence sICAS through anti-inflammatory and lipid responses and may also contribute to improving neurodegenerative diseases.

LBP an acute-phase protein predominantly synthesized by hepatocytes, has a molecular weight of 6×10^4^ and comprises an N-terminal and a C-terminal region (25). LBP serves two vital functions: it enhances lipopolysaccharide (LPS) biological activity and amplifies the LPS-mediated inflammatory response; additionally, LPS can be cleared *in vivo* by binding to high-density lipoprotein (HDL) via LBP and subsequently translocating to the liver. Several studies have posited (26, 27) that LBP’s primary *in vivo* function is to deliver LPS to CD14 receptors on monocyte macrophages and neutrophils’ surfaces, forming the LPS/LBP/CDl4 complex. This activates the inflammatory signaling pathway, amplifies the LPS-mediated inflammatory response, and triggers a cascade of responses leading to various pathophysiological processes. This study reveals that LBP, in the XXZ group of acute ischemic stroke patients, is mainly associated with “inflammatory response,” “response to lipopolysaccharide,” and “response to lipid.” Reducing LBP expression may be pivotal for preventing and treating sICAS, warranting further investigation.

CTSG a member of the serine isoform of the histone family, originates from neutrophils, monocytes, and mast cells. It plays a role in regulating the functional state of immune cells. Neutrophil infiltration, a prominent feature of acute inflammatory diseases, is largely influenced by CTSG (28, 29). CTSG is significant in the pathology of anti-neutrophil cytoplasmic antibody-associated small-vessel vasculitis and acts as a potent platelet activator and thrombosis modulator (30). This study revealed that in the XXZ group of patients with acute ischemic stroke, CTSG was primarily associated with “response to lipopolysaccharide” and “response to lipid.” Consequently, inhibiting the expression of A2M, CTSG, and LBP may not only protect against sICAS but also mitigate the related inflammatory response, thereby presenting a novel target for the prevention and treatment of inflammatory diseases.

Cystatin C (Cys C), belonging to the cysteine protease inhibitor super-family 2, is encoded by the CST3 gene located on the short arm of human chromosome 20, region 13, band 2. Numerous studies have demonstrated (31, 32) that Cys C and its degradation products influence the phagocytosis and chemotaxis of neutrophils, participate in the inflammatory response, and contribute to the dynamic balance between extracellular matrix production and degradation. The development of atherosclerosis is closely linked to extracellular matrix degradation and vascular wall remodeling (33–36). This study indicated that CST3 in the XXZ group of acute ischemic stroke patients was mainly associated with “response to lipid,” aligning with previous studies linking Cys C to atherosclerosis.

FABP1 plays a crucial role in the transport and metabolism of free fatty acids in liver cells (37). The human FABP1 gene is situated in the chromosome 2p11.2 region (37, 38). Mutations in FABP1 T94A may influence the uptake, transport, and metabolism of free fatty acids, thereby affecting blood lipid levels and potentially leading to lipid metabolism disorders (39). This study demonstrated that FABP1 in the XXZ group of patients with acute ischemic stroke was predominantly associated with the “cellular lipid metabolic process” and “response to lipid,” aligning with both domestic and international research on FABP1 and dyslipidemia. Thus, increased expression of Cys C and FABP1 may impact sICAS through the lipid response. In summary, this research uncovers, for the first time, alterations in plasma proteins in acute ischemic stroke patients with sICAS. Five proteins (A2M, LBP, CTSG, CST3, FABP1) are identified as contributing to the development of atherosclerotic stenosis in intracranial arteries through their roles in lipid metabolic pathways and inflammatory responses. Biomarkers, due to their rapid, effective, and non-invasive nature, are becoming increasingly prevalent in clinical research. Hence, in clinical diagnosis and treatment, serum marker tests for A2M, LBP, CTSG, CST3, and FABP1 should be prioritized in patients with a high suspicion of stroke for early detection and timely intervention. This study acknowledges limitations such as a small sample size and geographical homogeneity, which can lead to representativeness errors in statistical estimates. Future research should expand the sample size, diversify geographical case collections, and conduct more comprehensive investigations. Identifying biomarkers through differential changes in peripheral blood plasma proteins can lay the foundation for diagnosing sICAS, treating stroke, and preventing stroke recurrence. This study can contribute to the formulation of personalized stroke diagnosis and treatment protocols and foster the development of drugs for preventing acute ischemic stroke recurrence through a combination of Chinese and Western medicine, thereby advancing modern precision medicine.

## Data availability statement

The datasets presented in this study can be found in online repositories. The names of the repository/repositories and accession number(s) can be found at: http://www.proteomexchange.org/, PXD044579.

## Ethics statement

The studies involving humans were approved by Ethics Committee of the Institute of Clinical Basic Medicine of Chinese Medicine. The studies were conducted in accordance with the local legislation and institutional requirements. The participants provided their written informed consent to participate in this study.

## Author contributions

YD: Formal analysis, Funding acquisition, Project administration, Resources, Software, Validation, Visualization, Writing – original draft. JL: Data curation, Formal analysis, Methodology, Software, Supervision, Writing – original draft. HS: Conceptualization, Formal analysis, Investigation, Project administration, Resources, Software, Validation, Writing – original draft. SY: Investigation, Resources, Software, Supervision, Visualization, Writing – original draft. XW: Data curation, Resources, Software, Writing – original draft. DZ: Funding acquisition, Resources, Writing – review & editing.
